# Acute Q Fever with Atrioventricular Block, Israel

**DOI:** 10.3201/eid2809.212565

**Published:** 2022-09

**Authors:** Karawan Badarni, Miry Blich, Yafit Atiya-Nasagi, Nesrin Ghanem-zoabi

**Affiliations:** Rambam Health Care Campus, Haifa, Israel (K. Badarni, M. Blich, N. Ghanem-zoabi);; The Ruth and Bruce Rappaport Faculty of Medicine of Technion, Haifa (M. Blich, N. Ghanem-zoabi);; Israel Institute for Biological Research, Ness-Ziona, Israel (Y. Atiya-Nasagi)

**Keywords:** Q fever, *Coxiella burnetii*, bacteria, atrioventricular block, zoonoses, Israel

## Abstract

Cardiac involvement in acute Q fever is rare. We report 2 cases of an advanced atrioventricular block in young adult patients in Israel who sought care for acute Q fever without evidence of myocarditis. Q fever should be suspected in unexplained conduction abnormalities, especially in febrile young patients residing in disease-endemic areas.

Q fever is a zoonosis caused by *Coxiella burnetii* bacteria; the main route of infection is through inhalation of infected aerosols (*1*). Acute Q fever is mainly a self-limited influenza-like illness but may manifest as pneumonia or hepatitis. Less common manifestations involve different organs of the nervous, cardiovascular, skin, gastrointestinal, and hematopoietic systems (*2*). Cardiac involvement in Q fever is usually observed in the chronic form and manifests as endocarditis, aortitis, and vascular aneurysm infection. Less common cardiovascular manifestations in acute Q fever include myocarditis, pericarditis, and acute endocarditis (*2*). We report 2 patients in Israel who had acute Q fever and advanced atrioventricular block as the cardiac manifestation.

Patient 1 was a 48-year-old man was admitted to an intensive cardiac care unit (ICCU) for dizziness and electrocardiogram (EKG) abnormalities. He had type 2 diabetes mellitus and hypertension. Ten days before admission, he had fever and myalgia; his symptoms lasted for 1 week and resolved spontaneously. Three days later, he experienced dizziness; he sought care, and an EKG showed a new atrioventricular block.

At admission, the patient’s vital signs were within reference ranges except for bradycardia (37 bpm); results of a physical examination was unremarkable. Complete blood count showed decreased hemoglobin (12.5 g/dL) and elevated C-reactive protein. Liver enzyme, cardiac troponin, and coagulation test results were all within reference ranges, and blood cultures were negative. Serology testing for brucellosis was negative. Chest radiograph showed no pathology. His EKG showed a complete atrioventricular block with a ventricular escape rate of 35 bpm and a QRS wave duration of 140 ms (Figure 1). A cardiac evaluation including resting EKG with standard and high leads, 12-lead Holter EKG, and echocardiogram all excluded a channelopathy or cardiomyopathy; cardiac stress testing excluded ischemia. Fluorodeoxyglucose positron emission computed tomography (FDG/PET-CT) showed no pathological uptake in the myocardium or elsewhere in the body. Because of his symptomatic atrioventricular block, a permanent pacemaker was implanted.

**Figure 1:**
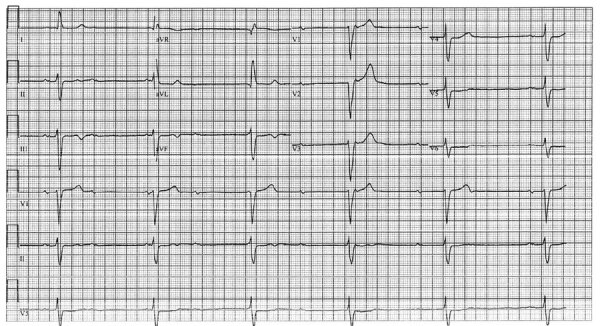
Electrocardiogram of a a 48-year-old patient in Israel with Q fever, showing a complete atrioventricular block with ventricular escape rate of 35 bpm and a QRS duration of 140 ms.

The patient had no familial history of cardiac conduction defects or cardiomyopathy. We suspected infectious etiology because of his history of a febrile illness preceding the atrioventricular block occurrence. This patient lived in northern Israel, where Q fever is endemic, and worked in a slaughterhouse. Serology testing for *C. burnetii* demonstrated positive phase II IgM, phase II IgG with a titer of 1:100, and negative phase I IgG. PCR testing for *C. burnetii* (targeting insertion sequence 1111) in serum returned negative results. Repeated serologic testing 4 weeks later demonstrated titers of phase II IgG had increased to 3,200. We used an in-house indirect immunofluorescence assay with a cutoff of 1:100 to diagnose definitive acute Q fever and treated the patient with doxycycline (100 mg 2×/d for 2 weeks). Pacemaker testing 5 months and 12 months later showed no restoration of normal conduction. Repeated serologic testing showed no evidence for progression to chronic disease, and repeated echocardiography and FDG/PET-CT showed no focal infection.

Patient 2 was an otherwise healthy 23-year-old man referred to the ICCU for recurrent episodes of syncope and EKG abnormalities. At admission, his vital signs were within reference ranges except for bradycardia (35 bpm). Results of a physical examination were unremarkable. Results of blood tests including complete blood count, coagulation tests, liver enzymes, C-reactive protein, and cardiac troponin were all within reference ranges. Chest radiograph showed no pathology, but EKG showed a left bundle branch block with sinus bradycardia 35 bpm (Figure 2). Electrophysiologic study demonstrated an infranodal dysfunction. Echocardiography showed a normal heart function; cardiac stress testing excluded ischemia. Cardiac magnetic resonance imaging results ruled out myocardial inflammation or infiltration process. FDG/PET-CT revealed no pathological reuptake. The patient’s family history was unremarkable for premature sudden cardiac death, cardiomyopathy, and inherited arrythmias. Other systemic causes for atrioventricular block–like hypothyroidism and hemochromatosis were ruled out. Serologic test results for syphilis and toxoplasmosis were negative. 

**Figure 2:**
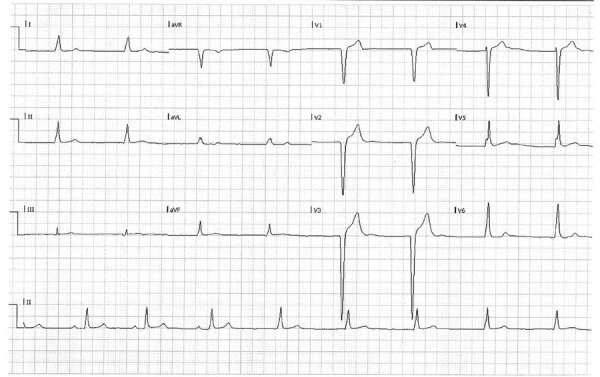
Electrocardiogram of a 23-year-old patient in Israel with Q fever, showing a left bundle branch block with sinus bradycardia of 35 bpm.

The patient, who lived in northern Israel, denied direct contact with livestock animals; however, he raised and bred horses. Serologic testing for *C. burnetii* was positive for phase II IgM and IgG, with titers of 1:400 and 1:800 taken at admission and 4 weeks later. Results of testing for phase I IgM and IgG and *C. burnetii* PCR in serum were all negative.

We treated the patient with doxycycline (100 mg 2×/d for 2 weeks). After 10 days of observation, his heart returned to normal sinus rhythm, and no permanent pacing was indicated. The patient was discharged, but no follow-up was completed.

The diagnosis of acute Q fever in patient 1 was considered definitive on the basis of a 4-fold increase in antibody titer in 2 consecutive serum samples (*3*). Patient 2 did not have classic symptoms of Q fever, but his serology profile was consistent with probable acute infection (3). The high titer of phase II IgG in the presence of positive phase II IgM titer is highly suggestive of a recent acute infection (*4*). PCR testing for *C. burnetti* was negative, consistent with the acute setting in which the bacteremia is short and usually precedes antibody formation (*5*). After thorough cardiac evaluation of each patient, we found no alternative diagnosis explaining an advanced atrioventricular block at their young ages.

## Conclusions

Myocardial involvement in acute Q fever is rare and reported among 0.5%–1% of cases (*2*,*6*). Cardiac complications include myocarditis, pericarditis, and acute endocarditis (*2*). Patients with myocarditis often seek care for shortness of breath, chest pain, or heart failure. Cardiac enzymes are usually elevated with or without EKG changes; ST-T segment abnormalities and T-wave inversion are the most frequent. None of these features existed in these patients. The reported course of Q fever myocarditis is prolonged and linked with high mortality rates (*6*).

Acute Q fever endocarditis was suggested as an autoimmune complication in 9/759 patients (1.2%) infected with *C. burnetii*; these patients had valve vegetations detected by echocardiography and positive anticardiolipin antibodies (*7*). The presence of antiphospholipid autoantibodies is known to lead to cardiac damage causing different manifestations, such as coronary artery disease, valve abnormalities, and myocardial dysfunction (*8*). Although rarely described, atrioventricular block can be complicated by these antibodies (*9*). This complication was described in other autoimmune disease, such as systemic lupus erythematosus (*10*). We did not perform evaluation of these patients for anticardiolipin antibodies. However, imaging results showed no vegetations or valve thickening.

Few infectious diseases have a predilection for the conduction system; the most well-recognized one is Lyme disease, in which the atrioventricular block is transient and can be the only cardiac manifestation, as we observed in patient 2. Animal studies in mice infected with *Borrelia burgdorferi*, the causative bacterium of Lyme disease*,* have demonstrated a higher burden of infection within the heart connective tissue (*11*). Chagas disease, a parasitic tropical disease caused by infection with the parasite *Trypanosoma cruzi*, may manifest with various conduction abnormalities usually accompanying myocarditis (*12*).

The pathophysiology in which the cardiac abnormality was persistent in patient 1 and transient in patient 2 is not fully understood. We hypothesize that either a direct damage of *C. burnetii* to the conduction system or, more likely, an indirect damage through an inflammatory process with or without autoantibodies may have occurred in both patients. This damage was theoretically more critical in patient 1, whose disease course was more severe than that of patient 2 and led to an irreversible atrioventricular block. In areas to which Q fever is endemic, *C. burnetii* should be considered a possible etiology for unexplained conduction disorders in young adults.
